# Myopic Control Using Defocus-Incorporated Multiple Segments Spectacle Lenses Plus Low-Concentration Atropine in Patients With Fast Myopia Progression: A Retrospective Cohort Study

**DOI:** 10.7759/cureus.92934

**Published:** 2025-09-22

**Authors:** Chia-Yi Lee, Shun-Fa Yang, Ching-Yao Huang, Chao Kai Chang, Jing-Yang Huang, Yu-Ling Chang

**Affiliations:** 1 Institute of Medicine, Chung Shan Medical University, Taichung, TWN; 2 Department of Optometry, Da-Yeh University, Changhua, TWN; 3 Department of Ophthalmology, Nobel Eye Institute, Taipei, TWN; 4 Department of Medical Research, Chung Shan Medical University Hospital, Taichung, TWN; 5 Department of Pediatric Medicine, Cathay General Hospital, Taipei, TWN

**Keywords:** atropine, axial length, defocus incorporated multiple segments, myopia, myopia progression

## Abstract

Purpose

The aim of this study is to evaluate the myopic control effect between defocus-incorporated multiple segments (DIMS) spectacle lenses plus 0.01% atropine (ATR), and 0.125% ATR, for patients with fast myopia progression.

Methods

A retrospective cohort study was conducted, and patients who had experienced myopic progression of more than -1.00 diopter (D) within a one-year period were enrolled. Then, the patients were divided according to the management they received. A total of 53 and 46 eyes were assigned to the ATR and DIMS groups, respectively. The primary outcomes were the progression of spherical equivalent refraction (SER) and axial length (AXL). The independent t-test and generalized linear model were used for statistical analysis.

Results

After the one-year follow-up period, the progression of SER was significantly greater in the ATR group (0.31 ± 0.11 D) than in the DIMS group (0.10 ± 0.07 D) (p < 0.001). On the other hand, the elongation of AXL was significantly larger in the ATR group (0.12 ± 0.06 mm) than in the DIMS group (0.03 ± 0.02 mm) (p < 0.001) at the final visit. In addition, the ATR group with low- or moderate-baseline myopia revealed more significant SER and AXL progression than the DIMS group (all p < 0.001). A young age at onset was correlated with greater SER and AXL progression in the ATR group (both p < 0.05). Still, no pre-treatment parameter was associated with increased SER or AXL progression in the DIMS group (all p > 0.05).

Conclusions

DIMS spectacle lenses plus 0.01% ATR management showed greater efficacy for SER and AXL control than 0.125% ATR management in the fast myopia progression population.

## Introduction

Myopia is a growing health issue in many countries, with its prevalence reaching over 80% in Asian communities [1]. Myopia development occurs as a result of steep corneal curvature or a long axial length (AXL), with acquisition of the latter accounting for most cases of myopia [2,3]. Patients with high levels of myopia, most commonly defined as a spherical equivalent refraction (SER) greater than -6.00 diopters (D), are at higher risk of retinal, retinal pigment epithelium, and optic nerve changes [4]. Some complications of myopia, such as retinal detachment and myopic maculopathy, can lead to permanently impaired visual acuity [3].

Due to the influence of myopia on visual acuity and ocular health, several methods have been introduced to control its progression [5,6]. Atropine (ATR) eyedrops were the first and most widely applied treatment for myopia worldwide [7]. In a previous study, the administration of ATR was found to significantly reduce the progression of both SER and AXL [8,9]. In addition to ATR, orthokeratology contact lenses and dual-focus soft contact lenses have also been applied for myopia control, with good efficacy [1,10]. Recently, the use of defocus-incorporated multiple segments (DIMS) spectacle lenses has demonstrated a greater myopia control effect than that observed in the non-intervention group [11], and DIMS spectacle lenses and orthokeratology contact lenses have achieved similar levels of efficacy in controlling myopia [11,12].

A previous review article demonstrated similar efficacy, in terms of myopic control effects, between DIMS spectacle lenses and high-concentration ATR [12]. In addition, the combined usage of DIMS spectacle lenses and ATR is superior to DIMS spectacle lenses or ATR monotherapy [13,14]. However, few studies have evaluated the myopic control effect of DIMS spectacle lenses used alongside low-concentration ATR compared to high-concentration ATR. Furthermore, some patients experience rapidly progressing myopia of up to -1.00 D per year, and suitable methods for managing such cases still require further evaluation.

The objective of this study is to investigate the effect of DIMS spectacle lenses plus low-concentration ATR versus high-concentration ATR on controlling fast myopia progression, with the enrollment of several predictors.

## Materials and methods

Ethics declaration

All procedures performed in this study complied with the Declaration of Helsinki (1964) and its following amendments. This study was authorized by the Institutional Review Board of the National Changhua University of Education (project code: NCUEREC-112-071; date of approval: September 27, 2023). The requirement to obtain written informed consent was waived by the National Changhua University of Education due to the retrospective design of the study.

Patient selection

A retrospective cohort study was conducted by the Nobel Eye Institute, Taipei, Taiwan, which is a group of clinics composed of more than 20 branches located throughout the Taiwan region. Patients with the following characteristics were selected for this study: (1) age ranging from 6 to 15 years at the index date; (2) a fast myopic progression of -1.00 D or more identified within one year at any branch of the Nobel Eye Institute; (3) use of only 0.01% ATR for myopia control during the fast myopic progression period; and (4) participation in follow-up at any branch of the Nobel Eye Institute for at least one year after the fast myopia progression. The index date was set as the date that fast myopic progression was recorded. In addition, the following exclusion criteria were adopted to standardize the ocular condition of the study population: (1) corrected distance visual acuity (CDVA) less than 20/25 on a Snellen chart; (2) pre-existing ocular disease, including (but not limited to) corneal opacity, congenital glaucoma, congenital cataract, retinopathy of prematurity, optic nerve atrophy, ptosis, and microphthalmos; (3) high myopia, defined by a SER of more than -6.00 D at initial presentation; and (4) inability to visit the participating clinic at least once every two months. Next, the study population was further divided into two groups: (1) the DIMS group, who received 0.01% ATR plus DIMS spectacle lenses (MiyOSMART, Hoya, Tokyo, Japan) after the index date for one year; and (2) the ATR group, who were prescribed 0.125% ATR and single-vision spectacle lenses after the index date for one year. Patients who received different treatments were excluded from the study. Both groups were instructed to wear their spectacle lenses for at least 10 hours per day, and a “spectacle holiday” was not recommended by the physician. The time of ATR administration was 21:00 for all patients, and there was no “ATR holiday.” In addition, only the right eye of each patient was included in this study.

In summary, this retrospective cohort study selected patients who experienced fast myopic progression and divided them into different groups according to the myopia control methods they used. After the entire process, a total of 53 and 46 eyes were included in the ATR and DIMS groups, respectively.

Myopia examination

The baseline records (at the index date) of each child, including their age, sex, CDVA, sphere degree, cylinder degree, keratometry (corneal curvature), and AXL, were taken from the medical records of the Nobel Eye Institute. The primary outcomes (myopic indices) in this study were the amount of SER progression and AXL elongation after a one-year period. Cycloplegic SER and AXL were obtained using an autorefractor (KR-8900, Topcon, Tokyo, Japan) and a biometry device (IOL Master 500, Carl Zeiss Meditec, Jena, Germany), respectively. In addition, baseline keratometry was measured using a topographic machine (TMS-5, Tomey Corporation, Nagoya, Japan). Cycloplegic SER, involving both the sphere and cylinder degrees, was measured three times, and the average value was used in this study. For cycloplegia, topical tropicamide (Better Eye Drop, Aseptic Innovative Medicine Co., Ltd., Taoyuan, Taiwan) was administered at least three times, followed by an examination of the pupil diameter. The SER measurement was completed when the pupil diameter exceeded 8 mm. SER was defined as the sphere power plus half of the cylinder power. Regarding the follow-up procedure, the children were asked to visit the clinic every one to two months, and SER and AXL measurements were taken at each visit. The cycloplegic SER and AXL values before myopia intervention and one year after intervention in both groups were recorded and subsequently analyzed.

Statistical analysis

IBM SPSS Statistics for Windows, Version 20 (Released 2011; IBM Corp., Armonk, NY, USA), was used for our statistical analyses. The Shapiro-Wilk test was utilized to examine the normality of the two study groups, and both groups showed normal distributions (p > 0.05). Descriptive analysis represented the baseline conditions of the two groups. The independent t-test and Chi-square test were applied to compare baseline conditions between the two groups, depending on the characteristics of each parameter. The independent t-test was also used to compare SER and AXL progression between the two groups after a one-year interval. Next, the study populations were divided into patients with low baseline myopia (< -1.00 D one year before the index date) and moderate baseline myopia (> -1.00 D one year before the index date), and myopic progression in patients with different initial myopia baselines was compared via an independent t-test. Subsequently, the generalized linear model was used to analyze the correlation between myopic progression (i.e., SER progression and AXL elongation) and several covariates, including young initial age (less than 10 years), male sex, and steep keratometry (more than 45.00 D). The odds ratio (OR) and 95% confidence interval (CI) of each parameter for myopia progression were determined. Statistical significance was set at p < 0.05, and a p-value lower than 0.001 was reported as p < 0.001.

## Results

The baseline conditions of the ATR and DIMS groups are listed in Table 1. The mean initial age was 10.23 ± 1.36 and 10.11 ± 1.29 in the ATR and DIMS groups, respectively. The age difference between the two groups did not reach statistical significance (p = 0.655). In addition, the sex distributions between the two groups were also similar (p = 0.903). Regarding the ocular parameters, CDVA and keratometry did not exhibit significant differences between the two groups (both p > 0.05). For the myopic indices, the sphere degree, cylinder degree, SER, and AXL showed similar values between the two groups (all p > 0.05) (Table 1).

**Table 1:** Baseline characteristics between the two groups. An independent t-test and a Chi-square test were used for the statistical analysis. ATR: atropine; AXL: axial elongation; CDVA: corrected distance visual acuity; DIMS: defocus-incorporated multiple segments; N: number; SER: spherical equivalent refraction

Characteristics	ATR group (N = 53)	DIMS group (N = 46)	p
Age	10.23 ± 1.36	10.11 ± 1.29	0.655
Sex (male:female)	27:26	24:22	0.903
CDVA (LogMAR)	0.00 ± 0.00	0.00 ± 0.00	0.999
Sphere power	-1.62 ± 0.68	-1.70 ± 0.77	0.584
Cylinder power	-0.56 ± 0.24	-0.52 ± 0.27	0.437
SER	-1.90 ± 0.55	-1.96 ± 0.67	0.626
Keratometry	43.71 ± 1.21	43.44 ± 1.06	0.244
AXL	24.16 ± 0.57	24.20 ± 0.61	0.737

The initial SER was -1.90 ± 0.55 D and -1.96 ± 0.67 D in the ATR and DIMS groups, respectively. After the one-year follow-up period, the SER was -2.21 ± 0.63 D and -2.06 ± 0.69 D in the ATR and DIMS groups, respectively. The progression of SER was significantly greater in the ATR group (0.31 ± 0.11 D) than in the DIMS group (0.10 ± 0.07 D) (p < 0.001; Table 2). On the other hand, the baseline AXL was 24.16 ± 0.57 mm and 24.20 ± 0.61 mm in the ATR and DIMS groups, respectively. At the final visit, the AXL was 24.28 ± 0.68 mm and 24.23 ± 0.65 mm in the ATR and DIMS groups, respectively. The elongation of AXL was significantly larger in the ATR group (0.12 ± 0.06 mm) than in the DIMS group (0.03 ± 0.02 mm) (p < 0.001; Table 2).

**Table 2:** Progression of spherical equivalent refraction and axial length after the follow-up interval. An independent t-test was used for statistical analysis. * denotes a significant difference between the two groups. ATR: atropine; AXL: axial elongation; D: diopter; DIMS: defocus-incorporated multiple segments; N: number; SER: spherical equivalent refraction

Index	ATR group	DIMS group	p
SER (D)			
Pre-treatment	-1.90 ± 0.55	-1.96 ± 0.67	0.626
Post-treatment	-2.21 ± 0.63	-2.06 ± 0.69	0.261
Progression	0.31 ± 0.11	0.10 ± 0.07	<0.001*
AXL (mm)			
Pre-treatment	24.16 ± 0.57	24.20 ± 0.61	0.737
Post-treatment	24.28 ± 0.68	24.23 ± 0.65	0.710
Progression	0.12 ± 0.06	0.03 ± 0.02	<0.001*

Regarding patients with different characteristics, the ATR group with low-baseline myopia exhibited greater SER and AXL progression than the DIMS group with low-baseline myopia (both p < 0.001). Similarly, the ATR group with moderate-baseline myopia showed greater SER and AXL progression than the DIMS group with moderate-baseline myopia (both p < 0.001) (Table 3). On the other hand, a young initial age correlated with more significant SER and AXL progression in the ATR group (both p < 0.05) (Table 4). However, no pre-treatment parameter was associated with greater SER or AXL progression in the DIMS group (all p > 0.05) (Table 5).

**Table 3:** Myopia progression between the two groups with different baseline myopia degrees. An independent t-test was used for the statistical analysis. * denotes significant correlation between parameter and myopic progression. # denotes a significant difference value compared to the low-baseline myopia population (p < 0.05). ATR: atropine; DIMS: defocus-incorporated multiple segments; AXL: axial elongation; CI: confidence interval; OR: odds ratio; SER: spherical equivalent refraction

Myopia degree	ATR group	DIMS group	p
Low-baseline myopia			
SER progression	0.19 ± 0.09	0.09 ± 0.05	<0.001*
AXL progression	0.07 ± 0.05	0.03 ± 0.01	<0.001*
Moderate-baseline myopia			
SER progression	0.39 ± 0.16^#^	0.11 ± 0.10	<0.001*
AXL progression	0.15 ± 0.11^#^	0.03 ± 0.03	<0.001*

**Table 4:** Risk factors for myopic progression in the ATR group. A generalized linear model was used for the statistical analysis. * denotes significant correlation between parameter and myopic progression. AXL: axial elongation; CI: confidence interval; OR: odds ratio; SER: spherical equivalent refraction; ATR: atropine

Parameter	OR	95% CI	p
SER			
Young initial age	0.946	0.921-0.972	0.003*
Male sex	0.988	0.974-1.002	0.117
Steep keratometry	1.002	0.996-1.008	0.902
AXL			
Young initial age	0.959	0.932-0.986	0.009*
Male sex	0.994	0.985-1.003	0.106
Steep keratometry	1.001	0.998-1.004	0.964

**Table 5:** Risk factor for myopic progression in the DIMS group. A generalized linear model was used for the statistical analysis. AXL: axial elongation; CI: confidence interval; OR: odds ratio; SER: spherical equivalent refraction; DIMS: defocus-incorporated multiple segments

Parameter	OR	95% CI	p
SER			
Young initial age	0.993	0.982-1.005	0.279
Male sex	0.990	0.977-1.003	0.178
Steep keratometry	1.003	0.994-1.012	0.834
AXL			
Young initial age	0.996	0.989-1.004	0.336
Male sex	0.995	0.983-1.008	0.292
Steep keratometry	1.000	0.996-1.004	0.991

## Discussion

The SER progression and AXL elongation in the ATR group, using 0.125% ATR and single-vision spectacle lenses, were significantly higher than in the DIMS group, which used 0.01% ATR and DIMS spectacle lenses. In addition, the higher efficiency of myopia control in the DIMS group was observed in both the low-baseline myopia and moderate-baseline myopia populations. On the other hand, a young initial age was associated with SER and AXL progression in the ATR group, while no factor was found to influence myopic progression in the DIMS group.

Myopia, especially high levels of myopia, can contribute to several ocular diseases, according to the literature [2]. One example is myopic maculopathy, characterized by a rupture of Bruch’s membrane and the outer retinal layer [3]. In severe cases of myopic maculopathy, choroidal neovascularization may develop, and intravitreal injection of anti-vascular endothelial growth factor may be needed to restore visual acuity [2,3]. Even with prompt treatment, the prognosis of myopic maculopathy is guarded, and severe visual impairment may occur [15]. Retinal detachment is another serious comorbidity of myopia, especially in cases of high myopia [3]. Rhegmatogenous retinal detachment is more common in patients with higher myopia; a 200-fold higher risk of rhegmatogenous retinal detachment has been observed in the high-myopia population compared to the general population [2]. Surgical management techniques such as scleral buckling, pneumatic retinopexy, and trans pars plana vitrectomy may be warranted to treat rhegmatogenous retinal detachment, but postoperative visual recovery is not guaranteed [16]. In addition to retinal disorders, glaucoma is also a common disease in patients with high levels of myopia, and it can lead to irreversible visual impairment [3,17]. Accordingly, the prevention of myopia progression and the development of high myopia is important. To date, several tools have been established that can effectively control myopia. These include ATR, orthokeratology contact lenses, dual-focus soft contact lenses, and DIMS spectacle lenses [18]. Despite their efficacy in the general population, their effects on specific populations remain relatively unclear. We propose that a myopia control tool with a different design or mechanism may be more effective than a single therapy in vulnerable populations, and this hypothesis is supported by the results of this study.

The DIMS group demonstrated a better myopia control effect regarding SER progression and AXL elongation compared to the ATR group. In previous research, it was found that both DIMS spectacle lenses and 0.125% ATR can effectively control myopia progression [19,20]. In addition, DIMS plus low-concentration ATR is another myopia control intervention with high efficacy and relatively few side effects [21]. The myopia control effect of DIMS plus low-concentration ATR is numerically higher than that of low-concentration ATR alone in the general population, according to a previous study [22]. Still, there is scant research investigating the efficacy of DIMS plus low-concentration ATR and high-concentration ATR in specific populations. To our knowledge, the findings of this study may serve as a preliminary investigation illustrating the higher efficacy of DIMS plus 0.01% ATR on SER and AXL control compared to 0.125% ATR in patients with fast myopia progression. In addition, the baseline characteristics of the two groups were similar; thus, the influence of initial age or the degree of myopia on the myopia control effect may not be prominent. The initial age, initial SER, and initial AXL between the two groups demonstrated no significant differences. Although we did not strictly calculate the hours of near work, this value was estimated to be around three to four hours after school in both groups, according to their parents. Still, further study to evaluate the influence of these parameters on myopia progression in a similar population is warranted. Additionally, the protocols for ATR administration and DIMS spectacle lens usage were the same, so patients received consistent management. Consequently, the application of DIMS spectacle lenses plus 0.01% ATR may indeed provide a greater myopia control effect than 0.125% ATR plus single-vision spectacle lenses in patients with fast myopia progression. The degree of SER progression was about -0.30 and -0.10 D in the ATR and DIMS groups, respectively, which is much lower than the 1.00 D progression reported for the two groups in the previous year. Although the DIMS group exhibited more effective myopia control than the ATR group, the results may indicate that both myopia control strategies used by our institution were reasonable and effective.

Regarding the factors that may have influenced the myopic control effect in our population, patients with low or moderate degrees of myopia would benefit from using DIMS spectacle lenses plus 0.01% ATR management, as opposed to 0.125% ATR management. A higher baseline degree of myopia could be associated with significant myopia progression, based on the preceding publication [23]. Hence, myopia may be more difficult to control in patients with a higher baseline degree of myopia. In our study, DIMS spectacle lenses plus 0.01% ATR management were found to achieve greater myopic control in both subgroups, which further reflects the efficacy of the technique. Although the differences in myopia control effects between the DIMS and ATR groups were significant in both myopia subgroups, the amount of SER and AXL progression in the two groups within the low-baseline myopia population was numerically closer than in the moderate-baseline myopia counterpart. This result may indicate that 0.125% ATR therapy might be applied in low-baseline myopia patients who experience fast myopia progression. A young initial age was also found to be associated with myopia progression in the ATR group, but not in the DIMS group. Young age is a known predisposing factor for myopia progression, as established in a previous study [24]. A high level of myopia is also more common in individuals who experience early-onset myopia [25]. Thus, the results of this study may support the high myopic control efficacy of DIMS spectacle lenses plus 0.01% ATR management, as young age does not appear to affect myopic progression in such a population. Male sex and steep keratometry were not identified as risk factors for myopic development in previous studies [3], and our results corresponded with the existing research findings.

Regarding the myopic control efficiency achieved by our institution, the SER progression after one year of myopic control management was -0.31 ± 0.11 D and -0.10 ± 0.07 D in the ATR and DIMS groups, respectively. In an earlier study, the SER progression recorded one year after 0.125% ATR application was approximately -0.05 to -0.33 D [26,27]. In another study that used DIMS spectacle lenses for myopic control, the one-year SER progression was -0.17 D [28]. As a result, the SER progression in our study population may be comparable to that reported in other cases. For AXL control, the AXL elongation was 0.12 ± 0.06 mm and 0.03 ± 0.02 mm in the ATR group and DIMS group, respectively. When we reviewed preceding publications, the AXL elongation in patients who received high-concentration ATR management was about 0.37 mm over one year [29]. In a previous study in which DIMS spectacle lenses were adopted for myopia control, the annual AXL elongation was about 0.10 mm [28]. The mean AXL elongation in our patients may be comparable to that in these other studies. If we compare the SER and AXL progression in this study to previous studies that applied other myopia control tools, the changes in SER and AXL appear small [29,30]. Consequently, the myopic control quality of our institution - considering both SER and AXL progression - may be considered acceptable.

This study has several limitations. Firstly, the retrospective nature of this study reduces the homogeneity of the study population. Although the baseline characteristics are similar, the actual usage of myopia control tools may differ under such conditions. In addition, patients did not see the same optometrist at every visit, and the data were collected from different clinics; thus, the visits may have differed somewhat, and the variability of treatment may be prominent. Furthermore, the small patient group used in this study - in which only 99 eyes were included - reduced the study’s statistical power, although normal distribution was found in both groups. Finally, the exact pupil diameter was not measured in this study; this can serve as an indicator of whether full cycloplegia has been achieved.

## Conclusions

In conclusion, the combined usage of DIMS spectacle lenses plus 0.01% ATR management demonstrated greater SER and AXL control effects than 0.125% ATR management in patients with fast myopia progression. Furthermore, the high efficiency of combined treatment was consistent for different degrees of myopia. Consequently, DIMS spectacle lenses plus 0.01% ATR management may be recommended for children with fast myopia progression. A further large-scale prospective study, examining the myopic control effect between DIMS spectacle lenses plus 0.01% ATR management and other myopic control tools, with near visual hygiene evaluation in patients experiencing fast myopia progression, is required.

## References

[1] Bullimore MA, Richdale K (2020). Myopia control 2020: where are we and where are we heading?. Ophthalmic Physiol Opt.

[2] Cooper J, Tkatchenko AV (2018). A review of current concepts of the etiology and treatment of myopia. Eye Contact Lens.

[3] Morgan IG, Ohno-Matsui K, Saw SM (2012). Myopia. Lancet.

[4] Flitcroft DI, He M, Jonas JB (2019). IMI - defining and classifying myopia: a proposed set of standards for clinical and epidemiologic studies. Invest Ophthalmol Vis Sci.

[5] Walline JJ (2016). Myopia control: a review. Eye Contact Lens.

[6] Leo SW (2017). Current approaches to myopia control. Curr Opin Ophthalmol.

[7] Tran HD, Tran YH, Tran TD, Jong M, Coroneo M, Sankaridurg P (2018). A review of myopia control with atropine. J Ocul Pharmacol Ther.

[8] Wu PC, Chuang MN, Choi J (2019). Update in myopia and treatment strategy of atropine use in myopia control. Eye (Lond).

[9] Shih YF, Chen CH, Chou AC, Ho TC, Lin LL, Hung PT (1999). Effects of different concentrations of atropine on controlling myopia in myopic children. J Ocul Pharmacol Ther.

[10] Bullimore MA, Johnson LA (2020). Overnight orthokeratology. Cont Lens Anterior Eye.

[11] Lam CS, Tang WC, Tse DY (2020). Defocus incorporated multiple segments (DIMS) spectacle lenses slow myopia progression: a 2-year randomised clinical trial. Br J Ophthalmol.

[12] Jonas JB, Ang M, Cho P (2021). IMI prevention of myopia and its progression. Invest Ophthalmol Vis Sci.

[13] Huang Z, Chen XF, He T, Tang Y, Du CX (2022). Synergistic effects of defocus-incorporated multiple segments and atropine in slowing the progression of myopia. Sci Rep.

[14] Nucci P, Lembo A, Schiavetti I, Shah R, Edgar DF, Evans BJ (2023). A comparison of myopia control in European children and adolescents with defocus incorporated multiple segments (DIMS) spectacles, atropine, and combined DIMS/atropine. PLoS One.

[15] Gómez-Resa M, Burés-Jelstrup A, Mateo C (2014). Myopic traction maculopathy. Dev Ophthalmol.

[16] Warren A, Wang DW, Lim JI (2023). Rhegmatogenous retinal detachment surgery: a review. Clin Exp Ophthalmol.

[17] Ma F, Dai J, Sun X (2014). Progress in understanding the association between high myopia and primary open-angle glaucoma. Clin Exp Ophthalmol.

[18] Shah R, Vlasak N, Evans BJ (2024). High myopia: reviews of myopia control strategies and myopia complications. Ophthalmic Physiol Opt.

[19] Chen ZR, Chen SC, Wan TY (2023). Treatment of myopia with atropine 0.125% once every night compared with atropine 0.125% every other night: a pilot study. J Clin Med.

[20] Lam CS, Tang WC, Zhang HY (2023). Long-term myopia control effect and safety in children wearing DIMS spectacle lenses for 6 years. Sci Rep.

[21] Lee CY, Yang SF, Chang YL, Huang JY, Lian IB, Chang CK (2025). The effect of defocus incorporated multiple segment spectacles' lenses combined with different concentrations atropine for myopia control. Sci Rep.

[22] Guemes-Villahoz N, Talavero González P, Porras-Ángel P (2025). Atropine and spectacle lens combination treatment (aspect): 12-month results of a randomised controlled trial for myopia control using a combination of defocus incorporated multiple segments (DIMS) lenses and 0.025% atropine. Br J Ophthalmol.

[23] Li SM, Wei S, Atchison DA (2022). Annual incidences and progressions of myopia and high myopia in Chinese schoolchildren based on a 5-year cohort study. Invest Ophthalmol Vis Sci.

[24] Jones-Jordan LA, Sinnott LT, Chu RH (2021). Myopia progression as a function of sex, age, and ethnicity. Invest Ophthalmol Vis Sci.

[25] Pärssinen O, Kauppinen M (2019). Risk factors for high myopia: a 22-year follow-up study from childhood to adulthood. Acta Ophthalmol.

[26] Kim DH, Han J, Park KA, Yang HK, Kim US, Kim SH, Paik HJ (2025). Therapeutic effect of topical 0.125% atropine in Korean myopic children: the real-world experience. Korean J Ophthalmol.

[27] Lee CY, Sun CC, Lin YF, Lin KK (2016). Effects of topical atropine on intraocular pressure and myopia progression: a prospective comparative study. BMC Ophthalmol.

[28] Lam CS, Tang WC, Lee PH, Zhang HY, Qi H, Hasegawa K, To CH (2022). Myopia control effect of defocus incorporated multiple segments (DIMS) spectacle lens in Chinese children: results of a 3-year follow-up study. Br J Ophthalmol.

[29] Lin HJ, Wan L, Tsai FJ, Tsai YY, Chen LA, Tsai AL, Huang YC (2014). Overnight orthokeratology is comparable with atropine in controlling myopia. BMC Ophthalmol.

[30] Chamberlain P, Peixoto-de-Matos SC, Logan NS, Ngo C, Jones D, Young G (2019). A 3-year randomized clinical trial of MiSight lenses for myopia control. Optom Vis Sci.

